# DISCORDANT CHRONIC CONDITIONS AND DEPRESSIVE SYMPTOMS AMONG MIDDLE-AGED AND OLDER COUPLES

**DOI:** 10.1093/geroni/igz038.226

**Published:** 2019-11-08

**Authors:** Courtney A Polenick, Kira S Birditt, Angela Turkelson, Benjamin Bugajski, Helen C Kales

**Affiliations:** University of Michigan, Ann Arbor, Michigan, United States

## Abstract

Discordant chronic conditions (i.e., those with competing management requirements) have adverse consequences for well-being, yet little is known about their implications among couples. We evaluated how depressive symptoms are linked to discordant conditions within individuals and between spouses across an 8-year period. The U.S. sample included 1,116 middle-aged and older couples from five waves (2006 – 2014) of the Health and Retirement Study. Longitudinal actor-partner interdependence models controlled for age, minority status, education, depressive symptoms in the previous wave, and each partner’s report of baseline marital quality and number of chronic conditions in each wave. Wives and husbands with their own discordant conditions reported higher depressive symptoms, and this association intensified over time. Over and above this link, husbands had higher depressive symptoms when there were discordant conditions between spouses. Both individual-level and couple-level discordant chronic conditions appear to have enduring implications for depressive symptoms in middle and later life.

